# Molecular Epidemiology of Enteric Viral Infections in Poultry Flocks in Southern Germany and the First Complete Genome Sequence of Avian *Sicinivirus*

**DOI:** 10.3390/ani16091331

**Published:** 2026-04-27

**Authors:** Ibrahim Moharam, Julia Brüggemann, Ferdinand Schmitt, Benjamin Schade, Brigitte Böhm, Eva Kappe, Franziska Emmrich, Fares Z. Najar, Fouad S. El-Mayet

**Affiliations:** 1 Bavarian Animal Health Service, 85586 Poing, Germanyferdinand.schmitt@tgd-bayern.de (F.S.);; 2 Department of Bird and Rabbit Medicine, Faculty of Veterinary Medicine, University of Sadat City, Minoufiya 32897, Egypt; 3 High Performance Computing Center (HPCC), Oklahoma State University, Stillwater, OK 74078, USA; fnajar@okstate.edu; 4 Department of Pathobiology, Oklahoma State University, Stillwater, OK 74078, USA; 5 Department of Virology, Faculty of Veterinary Medicine, Benha University, Benha 13511, Egypt

**Keywords:** molecular epidemiology, RT-PCR, NGS, Sicinivirus, chicken astrovirus

## Abstract

Enteric viral infections represent a major concern for poultry production, causing growth retardation, impaired feed conversion, and increased mortality, particularly in young birds. To investigate the involvement of RNA and DNA enteric viruses in flocks exhibiting growth problems, seven poultry farms in southern Germany, including broiler, pullet, and breeder operations, were examined for the presence of chicken astrovirus (CAstV), avian reovirus (ARV), and fowl adenovirus-1 (FAdV-1) by means of RT-PCR. All farms exhibited growth retardation, diarrhea, and enteritis-associated lesions. Histopathology revealed features of runting–stunting syndrome in most of the broiler farms and depletion of lymphatic tissue in most of the pullet farms. CAstV was detected in all flocks, ARV in six, and FAdV-1 in four farms. To further characterize the viral agents, metagenomic sequencing of cecal tonsils from one severely affected broiler flock confirmed the presence of a CAstV strain identical (100%) to CAV/Belgium/4134_001/2019. In addition, the complete genome of avian Sicinivirus was assembled for the first time in Germany, showing 96.8% nucleotide identity with a Dutch strain (Chicken/NLD/2019/V_M_056_picorna_2). These findings demonstrate the widespread circulation and co-infection of enteric viruses on German poultry farms and underline the transboundary nature of these infections, emphasizing the need for enhanced surveillance and biosecurity measures to mitigate their impact on poultry health and productivity.

## Simple Summary

Viral diseases of the intestine are a common yet often overlooked problem in poultry farming. They can result in poor growth, diarrhea, and increased mortality, particularly in young chickens. In this study, several poultry farms in southern Germany experiencing enteric disease outbreaks were investigated to determine the specific viruses involved. Our analysis revealed that many of the affected birds harbored several viruses simultaneously, including agents already known to be associated with enteric disease in chickens. On one farm, an avian *Sicinivirus* was characterized, and its complete genome was sequenced for the first time in Germany. This virus is closely related to a strain previously discovered in the Netherlands, suggesting potential cross-border transmission. These results demonstrate that intestinal viruses are widespread in poultry flocks and that different viruses can co-occur, contributing to a more complex disease outlook. A more comprehensive understanding of circulating viruses will help veterinarians and farmers to improve animal health, reduce losses, and strengthen disease prevention in poultry farming.

## 1. Introduction

Over the past five decades, the poultry industry has experienced remarkable growth, establishing itself as a crucial contributor to global food security and animal protein supply [1]. This expansion has been accompanied by increasing challenges related to infectious diseases, impacting bird health and production performance. Within this context, enteric diseases represent a major cause of economic losses due to impaired feed conversion, poor weight gain, and reduced flock uniformity [2]. Despite continuous improvements in management and biosecurity, the ubiquity and genetic diversity of enteric viruses make it virtually impossible to maintain commercial flocks free of infection. Moreover, factors such as nutritional imbalances, stress, and the presence of other enteric pathogens, including bacteria, protozoa, and parasites, can exacerbate clinical outcomes [2].

The intestinal ecosystem, comprising the microbiome and virome, plays a critical role in maintaining gut health and overall homeostasis. The complex microbial and viral interactions in the gastrointestinal tract complicate efforts to delineate the specific contribution of individual pathogens to disease [3,4]. In poultry, enteric viruses are often considered primary pathogens initiating intestinal damage, which subsequently facilitates bacterial colonization and secondary infections [5].

Although numerous research groups have explored the virome of humans and various animal species, the avian virome remains comparatively underexplored [6,7]. In particular, little is known about the viral composition of the intestinal tract in healthy broilers, as the majority of researchers have focused on symptomatic or wild birds [8]. Enteric disorders such as malabsorption syndrome (MAS) or runting–stunting syndrome (RSS) are complex conditions with multifactorial etiologies. They are characterized by uneven growth, poor feed efficiency, diarrhea, and increased mortality [2,9]. Viral interference with the immune system likely contributes to increased susceptibility to secondary infections [4].

Several viruses have been implicated in the etiology of RSS, including avian nephritis virus (ANV), chicken astrovirus (CAstV), parvovirus, rotavirus (RV), and avian reovirus (ARV) [10]. However, attempts to assign a single causative agent have been largely unsuccessful, emphasizing the polyetiological and synergistic nature of these infections. Earlier diagnostic efforts primarily relied on targeted PCR approaches focusing on selected viruses [11]. With the advent of next-generation sequencing (NGS), it has become possible to perform unbiased, high-throughput identification of diverse viral genomes in poultry samples [11,12,13]. Nevertheless, the lack of standardized in vitro propagation systems for many avian enteric viruses continues to limit biological characterization [14].

To date, most research groups have concentrated on enteric viruses in conventional broiler flocks, with limited information available regarding the enteric virome in pullets, laying hens, or alternative production systems such as organic broilers. In the present study, we therefore aim to investigate the presence and molecular characteristics of enteric viruses in different poultry species and production types in southern Germany. In addition to describing the clinical and pathological findings in affected flocks, we report the first complete genome sequence of avian Sicinivirus detected in Germany, providing insights into its phylogenetic relationship with strains previously reported in other European countries.

## 2. Materials and Methods

### 2.1. Farm History

In this study, seven poultry flocks from six different farms in southern Germany were examined, including three broiler flocks (two from the same farm during different production cycles), three pullet flocks (one organic and two conventional), and one conventional broiler breeder flock. Carcasses of deceased birds and birds exhibiting severe symptoms were humanely euthanized and collected during routine visits by the field veterinarian and transported under refrigeration to the diagnostic laboratories at the Bavarian Animal Health Service. Farm 1 was visited twice by the attending veterinarian. During these visits, samples were collected from the same flock at two different time points.

### 2.2. Sample Collection and Histopathology

Upon arrival, the deceased birds were dissected. Under aseptic conditions, samples were collected for bacteriological, histopathological, and molecular analyses. To perform histological examination, the following organ sets were collected: bursa cloacalis, kidneys, liver, small intestine (duodenum), pancreas, proventriculus, ventriculus, brain, and spleen. To conduct bacteriological analysis, liver and small intestine samples were cultured. From Farm 2, the yolk sac and spleen were also cultured. To perform virological examination (both PCR and NGS), cecal tonsils were used. In addition to cecal tonsils, thymus, spleen, and a bursa swab were collected from animals on Farm 5. Based on the number of birds dissected per farm, at least two sets from each organ were cultured or analysed. To perform histopathology, tissues were fixed in 10% neutral buffered formalin and processed using standard protocols. Four-micrometre tissue sections were stained with hematoxylin and eosin for examination under a light microscope.

### 2.3. Identification of Bacterial Species in Poultry Flocks

To perform bacteriological analysis, organ tissue and swab samples collected during necropsy were examined. First, the specimens were inoculated onto Columbia agar with 5% defibrinated sheep blood (Oxoid™, Thermo Fisher Scientific, Waltham, MA, USA) and eosin–methylene blue agar (Oxoid™, Thermo Fisher Scientific, USA). For the detection of anaerobic bacteria, additional cultures on glucose sheep blood agar based on the Zeissler method (Oxoid™, Thermo Fisher Scientific, Waltham, MA, USA) were performed. Plates were incubated for 22 ± 2 h at 37 ± 1 °C under aerobic or anaerobic conditions, respectively. Subsequently, bacterial growth was assessed morphologically. Isolates were initially evaluated based on phenotypic characteristics and subsequently identified by MALDI-TOF mass spectrometry (Bruker Corporation, Billerica, MA, USA). The assessment of clinical relevance was based on pathogenic potential, site of isolation and concordance with the clinical and pathological findings.

### 2.4. Validation of Identified Viral Species Using RT-PCR

Upon arrival at the laboratory, pooled swab samples were processed following a standardized protocol. Initially, samples were suspended in 1 mL of sterile phosphate-buffered saline (PBS) and subjected to three cycles of freezing and thawing to maximize the release of microbial nucleic acids. Subsequently, 200 μL of the processed material was utilized for the extraction of bacterial and viral nucleic acids. Total RNA was isolated from the supernatant of liver homogenate specimens using the QIAamp Viral RNA Mini Kit (Qiagen, GmbH, Hilden, Germany), according to the manufacturer’s instructions. Extracted RNA was subsequently reverse-transcribed into complementary DNA (cDNA) using the QuantiTect Reverse Transcription Kit (Qiagen, Qiagen, GmbH, Hilden, Germany), following the manufacturer’s protocol, and stored at −20 °C until further use.

To perform DNA extraction, total DNA was isolated from the processed material homogenate supernatants using the QIAamp DNA Mini Kit (Qiagen, Qiagen, GmbH, Hilden, Germany), according to the manufacturer’s instructions. The purified DNA was eluted in nuclease-free water and stored at −20 °C until use. For some bacteria, such as *Mycoplasma* spp. and Ornithobacterium rhinotracheale, Kytil easy-to-use kits, Kylt^®^ ORT, Kylt^®^ MGS Triplex (SAN Group Biotech Germany GmbH, Emstek, Germany), were used according to the manufacturer’s instructions. For the detection of chicken astrovirus (CAstV), avian reovirus (ARV), and fowl Adenovirus-1 (FAdV-1), the samples were sent to AniCon Laboratory (SAN Group Biotech Germany GmbH, Emstek, Germany), where RT-PCR was performed in accordance with the laboratory’s protocols.

### 2.5. Metagenome Sequencing, Contig Assembly, and Annotation for Identification of Viral Gene Sequences in Poultry Flocks

Total RNA extracted from the original cecal tonsil sample was reverse-transcribed into complementary DNA (cDNA). then subjected to whole-genome analysis using next-generation sequencing (NGS) at Eurofins Genomics Co., Ltd., Ebersberg Germany. Sequencing libraries were constructed according to the Illumina platform’s standard protocols, after which sequencing was performed on the Illumina NovaSeq 6000 system Illumina, Inc San Diego, CA, USA), generating paired-end reads (2 × 150 bp). Raw sequence data were quality-filtered and subsequently assembled into contigs using Megahit software v1.2.9 [15]. The assembled contigs were screened against viral sequences in the GenBank database via BLASTN, v 2.17.0 [16]. Contigs demonstrating homology to known chicken viral pathogens were selected for further examination.

### 2.6. Phylogenetic Analyses of Annotated Viral Gene Sequences

Phylogenetic analyses were performed using Molecular Evolutionary Genetics Analysis (MEGA), version X [17]. Phylogenetic trees were constructed by employing the neighbor-joining method, with bootstrap values calculated based on 1000 replicates to assess the robustness of the branches. The genetic relationship between avian Sicinivirus and chicken astroviruses (CAstVs) genes was evaluated in comparison to related viral sequences retrieved from GenBank. Pairwise genetic distances were calculated and subsequently visualized as heatmaps using R Studio (version 1.2.5019) using the function “pheatmap” as implemented in R (version 3.6.1) [18]. The antigenic determinants of the VP1 protein were explored using the approach of Kolaskar & Tongaonkar [19] with the IEDB Analysis Resource (Antibody epitope prediction (iedb.org)).

### 2.7. Submission of Viral Genome Sequences to the NCBI

The final genome sequences and their annotation were deposited in GenBank under the BioProject accession: PRJNA1298928, BioSample: SAMN50289189. Accession numbers: PX395415–PX395426 for CAstV and PX068467 for avian Sicinivirus.

## 3. Results

### 3.1. Clinical Findings

Seven poultry flocks from southern Germany were examined, including three broiler flocks (two from the same farm at different production cycles), three pullet flocks (one organic and two conventional), and one conventional broiler breeder flock. The flock sizes ranged from 5000 to 15,000 birds for the broilers, 6000 to 20,000 for the pullets, and 2000 for the breeders. The birds’ ages at sampling varied between 11 and 90 days (Table 1).

Clinically, broiler flocks exhibited growth retardation, poor uniformity, bloody diarrhea (three out of four), and respiratory distress, accompanied by increased mortality. Similar findings, including reduced performance and enteric disorders, were also observed in pullets and the breeder flock.

### 3.2. Identified Bacterial Species in Poultry Flocks and Their Molecular Characterization

Through classical bacteriology, *Escherichia coli* was identified in all samples and *Clostridium perfringens* in all but one flock (Table 2). In one organic broiler flock, *Mycoplasma gallisepticum*, *Mycoplasma synoviae*, and *Avibacterium paragallinarum* were additionally detected. *Mycoplasma synoviae* was also detected in the broiler breeder flock. *Ornithobacterium rhinotracheale* (ORT) was found in two flocks from the same farm but at different time points.

Coccidia oocysts were found in all but one pullet flock in combination with lesions in the ceca or small intestine (Table 3). There is a high likelihood that this outcome was the result of an infection with pathogenic coccidia species and not the detection of vaccine oocysts.

### 3.3. Validation of Identified Viral Species Using RT-PCR

RT-PCR screening revealed that chicken astrovirus (CAstV) was present in all seven flocks, with Ct values ranging between 20.7 and 36.4. Avian reovirus (ARV) was also detected in six flocks, exhibiting Ct values between 24.6 and 36.4. Fowl adenovirus (FAdV -1A–E) was found in two organic broiler flocks from the same farm and in the broiler breeder flock (Table 4). These results demonstrate a consistent co-circulation of multiple enteric viruses among the examined farms.

### 3.4. Pathological and Histopathological Findings

**Figure 1:**
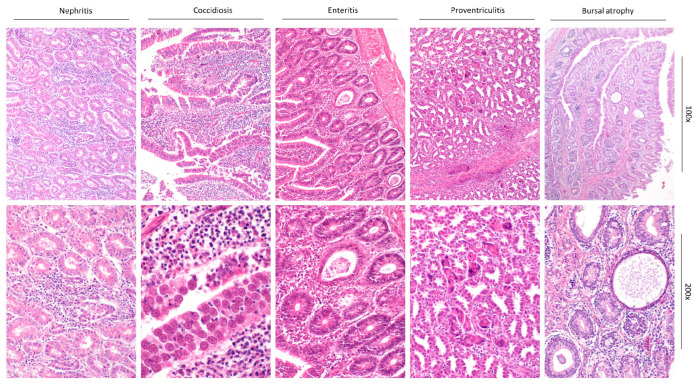
Representative pictures for histopathology of typical lesions in birds with runting–stunting syndrome (HE stain). From Left to Right: Nephritis with interstitial edema and infiltration of inflammatory cells accompanied by tubular degeneration. Coccidiosis of the small intestine with severe infiltration of inflammatory cells, necrotic debris, and coccidial oocysts. Enteritis with dilated crypts and interstitial infiltration of inflammatory cells. Proventriculitis with necrotic debris and dilation of glandular ducts. Bursal atrophy with vacuolization and inflammatory infiltrations.

Gross and histopathological examination revealed lesions compatible with enteric viral infections. Excluding one case, broilers exhibited features of runting–stunting syndrome in the intestine, such as villus atrophy, infiltration of the lamina propria with lymphocytes, and/or cystic dilatation of the crypts filled with cell debris. Only one broiler exhibited depletion of lymphatic tissues, which was not observed in any of the other birds. Additionally, one broiler case exhibited coccidiosis and non-suppurative interstitial nephritis (Figure 1).

The pullets, excluding one case, exhibited moderate-to-severe depletion of lymphatic tissues and coccidiosis. Features in the intestine characteristic of runting–stunting syndrome were only observed in one case. Additionally, two of the pullet cases exhibited nephromegaly with edema and non-suppurative, interstitial nephritis. Birds of the conventional pullet flock also exhibited proventriculitis with syncytial formation.

The organic pullet flock exhibited renal edema, with one conventional pullet flock exhibiting nephromegaly with interstitial nephritis, cecal coccidiosis, proventriculitis with syncytial formation, and fibrinous–granulomatous splenitis. The broiler breeder flock exhibited catarrhal tracheitis and sinusitis, proventricular folding, cecal coccidiosis, and mild interstitial salpingitis.

### 3.5. Identified Viral Species from Metagenomic Analysis and Their Molecular Characterization

Metagenomic sequencing of cecal tonsil samples from one severely affected broiler flock revealed the presence of multiple viral genome loads in the examined RNA.

**Figure 2:**
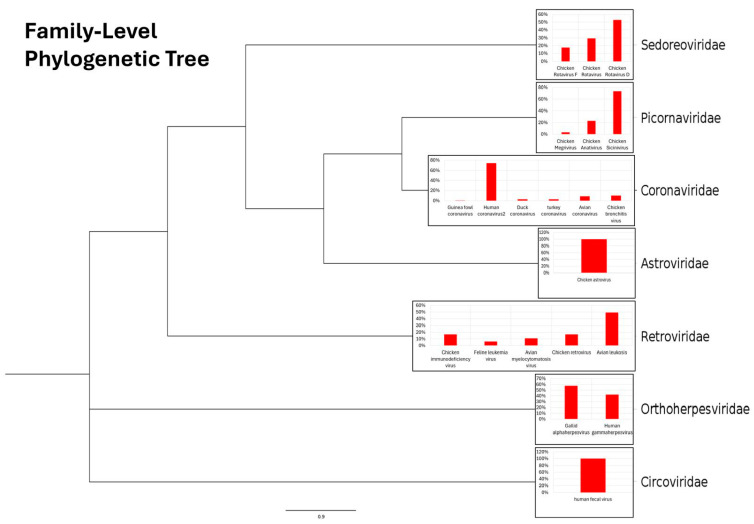
Taxonomic composition of viral sequences detected in a metagenome (BioProject accession: PRJNA1298928). Hierarchical clustering of detected viral taxa is shown as a dendrogram based on taxonomic relatedness. Viral sequences identified in the metagenomic dataset were classified and grouped by family. For each family, the relative abundances of individual viruses are displayed as inset bar plots (red bars), representing the percentage of total viral reads assigned to each detected virus within the dataset. The dendrogram illustrates the taxonomic relationships among viral families, whereas the accompanying bar charts quantify their relative representation in the sample. Relative abundance values were calculated as the proportion of reads assigned to each virus divided by the total number of viral reads in the metagenome, expressed as percentages.

The major viral families detected included *Coronaviridae* with 147 contigs, *Retroviridae* with 20 contigs, *Orthoherpesviridae* with 14 contigs, *Astroviridae* with 12 contigs, *Picornaviridae* with 8 contigs, *Sederoviridae* with 6 contigs, and lastly *Circoviridae* with 1 contig (Figure 2).

The complete genome sequence of an avian Sicinivirus has been characterized, representing the first report of this virus in Germany.

From 1344 reads with an average length of 150 bases, we obtained a total of 9733 nucleotides coding 10 genes representing the whole-genome sequence of the detected avian Sicinivirus, with the genome arrangement representing a typical Picornavirus genome (5UTR- VP0-VP3-VP1-2A-2B-2C-3A-3B-3C-3D-3 UTR) with a leader region from 1386 nucleotide/462 aa between the 5 UTR and the VP0 gene.

Detailed genome annotation revealed that the VP0 gene was located at nucleotides 2312–3328 (1017 nt), encoding a 339-amino acid protein. The VP3 gene spanned nucleotides 3329–3952 (624 nt) and encoded 208 amino acids; in comparison, the VP1 gene was positioned at nucleotides 3953–4891 (939 nt), encoding 313 amino acids.

Regarding the nonstructural proteins, the 2A gene comprised 483 nucleotides (nt 4904–5386) encoding 161 amino acids. The 2B gene was located at nucleotides 5387–5974 (588 nt) and encoded 196 amino acids. The 2C gene spanned nucleotides 5975–6994 (1020 nt), encoding 340 amino acids. The 3A gene was positioned at nucleotides 6995–7498 (504 nt) and encoded 168 amino acids. The 3B gene encompassed nucleotides 7499–7576 (78 nt), corresponding to 26 amino acids.

The 3C gene extended from nucleotides 7577–8101 (525 nt) and encoded 175 amino acids. Lastly, the 3D gene was located at nucleotides 8102–9520 (1419 nt), encoding a 473–amino acid protein.

**Figure 3:**
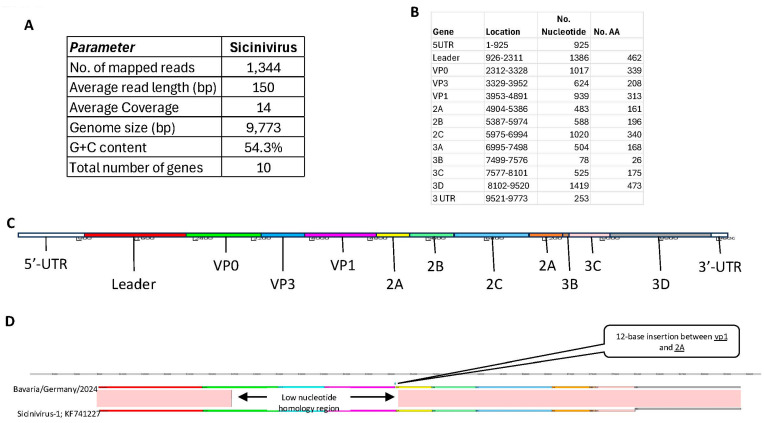
(**A**) Basic characteristics of the whole-genome sequencing (NGS), assembly, and annotation of Sicinivirus ME strain Bavaria/Germany/2024 (Accession No: PX068467) reported in the present study. (**B**–**D**) Schematic depicting the organization of the Sicinivirus ME strain.

The isolate was designated *Sicinivirus ME strain Bavaria/Germany/2024* (GenBank accession no. PX068467). Comparative analysis with the reference strain (GenBank accession no. KF741227) demonstrated greater sequence variability within the structural protein region than in the nonstructural region. Notably, a 12-nucleotide insertion was identified between the VP1 and 2A coding regions (Figure 3).

**Figure 4:**
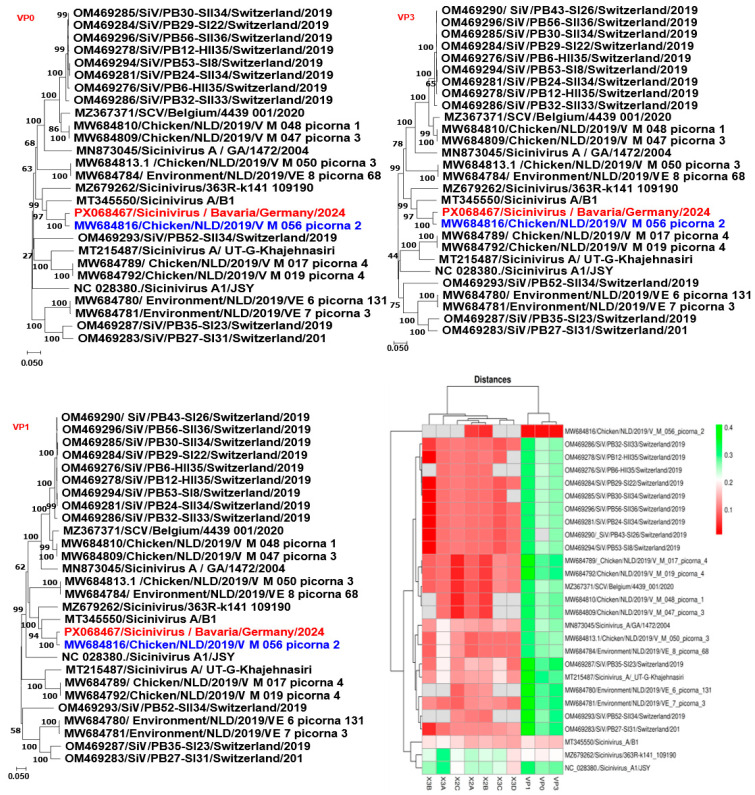
Phylogenetic analysis of Sicinivirus ME strain Bavaria/Germany/2024 based on nucleotide sequences of the complete genome (CG), viral protein 0 (VP0), VP3, and VP1 genes. Each tree was constructed using the neighbor-joining method, with branch lengths scaled to the number of substitutions per site (indicated by the scale bar). Bootstrap values (in percentages) from 1000 replicates are shown at the major branch points. The Sicinivirus ME strain Bavaria/Germany/2024 (highlighted in red) is positioned within the corresponding clades for each gene. GenBank accession numbers and strain designations are indicated for all sequences. The heatmap illustrates the nucleotide variability among strains, with clustering based on sequence similarity. Rows represent individual Sicinivirus strains (annotated with GenBank accession numbers and strain names), while columns correspond to specific nucleotide positions across the analyzed viral genes.

Genetic and phylogenetic analyses of Sicinivirus/Bavaria/Germany/2024 based on the structural proteins (VP0, VP3, and VP1) demonstrated clustering with three previously reported strains (MZ679262, MT345550, and MW684816). The highest sequence similarity (97% nucleotide identity) was observed with the avian Sicinivirus strain MW684816/Chicken/NLD/2019/V_M_056_picorna_2, which was characterized in the Netherlands in 2019. This close genetic relationship suggests potential transboundary circulation of Sicinivirus strains across Europe (Figure 4).

**Figure 5:**
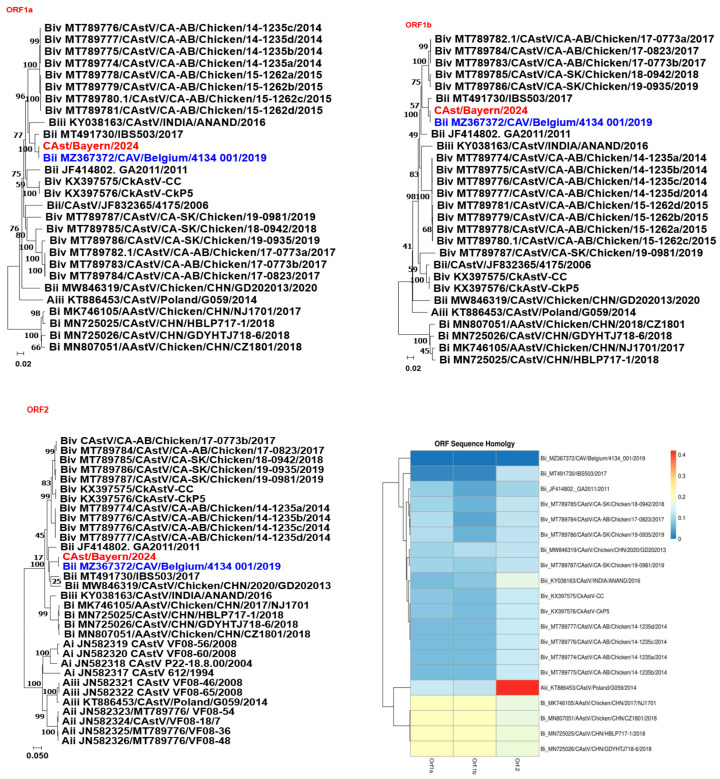
Phylogenetic analysis of CAstV strains based on nucleotide sequences of ORF1a, ORF1b, and ORF2 genes. Each tree was constructed using the neighbor-joining method, with branch lengths scaled to the number of substitutions per site (indicated by the scale bar). Bootstrap values (in percentages) from 1000 replicates are shown at the major branch points. The strain CAst/Bayern/2024 (highlighted in red) is positioned within the corresponding clades for each gene. GenBank accession numbers and strain designations are indicated for all sequences. The heatmap illustrates the nucleotide variability among strains, with clustering based on sequence similarity. Rows represent individual CAstV strains (annotated with GenBank accession numbers and strain names), while columns correspond to specific nucleotide positions across the analyzed viral genes.

CAstV was the only virus detected by means of both RT-PCR screening and NGS analysis, warranting further sequence characterization. Approximately 2900 nucleotides were assembled from 13 contigs (PX395415–PX395426). After gap removal and sequence refinement, 1502 nucleotides corresponding to ORF1a, 930 nucleotides corresponding to ORF1b, and 465 nucleotides of the capsid gene were obtained. Sequence analysis of all three regions revealed nearly 100% nucleotide identity with CAstV strain CAV/Belgium/4134_001/2019, which was identified in Belgium in 2019. Phylogenetic analysis further demonstrated that both viruses clustered within the CAstV group Bii lineage (Figure 5).

## 4. Discussion

The results of the present study provide molecular and metagenomic evidence for the widespread occurrence and co-circulation of multiple enteric viruses, including chicken astrovirus (CAstV), avian reovirus (ARV), and fowl adenovirus (FAdV), in poultry flocks in southern Germany. The detection of these viruses in broilers, pullets, and breeder flocks with enteric disorders and growth retardation confirms that mixed viral infections are common in commercial poultry production and may contribute synergistically to intestinal disease [11]. The relative viral abundance detected by means of metagenomics was consistent with previous observations in RSS-affected broiler flocks, where higher proportions of viral reads were found in clinically diseased birds compared with healthy controls [11]. However, as also noted by Devaney et al. [13], no single virus could be definitively associated with the observed clinical signs, supporting the multifactorial and polyetiological nature of enteric syndromes such as runting–stunting syndrome (RSS) and malabsorption syndrome (MAS). It should be noted that numerous viral families can be detected in the intestines of poultry, although not all of them have been proven to be pathogenic [8,13,20,21,22]. For example, chicken astroviruses are described as causing disease in chicks, but they are also found in adult chickens [23,24]. The situation is similar with reoviruses, which can be regularly detected in clinically healthy animals both serologically and via PCR or in culture [4,25,26,27]. For this reason, such findings should always be interpreted with due caution and in consideration of clinical and pathological findings.

In accordance with previous studies, the intestinal virome of healthy chickens is composed of a complex community of DNA and RNA viruses, including members of the *Adenoviridae*, *Caliciviridae*, *Circoviridae*, *Parvoviridae*, *Picobirnaviridae*, *Astroviridae*, and *Reoviridae* families [13,20]. In contrast, samples from clinically affected birds often exhibit a broader range and higher abundance of enteric viruses [13,14,28]. Our findings support the view that the virome composition alone does not determine disease outcome; rather, the interplay between host, pathogen, and environmental factors modulates the clinical expression of disease.

Through metagenomic analysis, both CAstV and avian Sicinivirus were identified as predominant RNA viruses in one severely affected broiler flock. The complete genomes of the Sicinivirus strain exhibited a high nucleotide identity (96.8%) to a Dutch strain, suggesting transboundary circulation of this virus in Europe. In addition, the CAstV was identical for the Belgian viruses, further substantiating the same argument. Although Sicinivirus has previously been detected in both asymptomatic and diseased chickens [11], its pathogenic potential remains unclear and warrants further studies on the pathogenicity of such viruses. The presence of almost identical strains in both healthy and affected birds, as also observed for CAstV and ANV, could indicate that these viruses may represent part of the normal intestinal virome rather than being direct causative agents of disease [11]. Alternatively, these viruses may exert pathogenic effects only under complex infections with other viruses, bacteria, and parasites. In contrast, the pathogenic effects of CAstV and ANV viruses have been demonstrated in numerous studies, as noted in the review by Smyth 2017 [29]; however, birds that appear clinically healthy at the time of examination may still harbor subclinical or latent infections.

The dynamic development of the intestinal RNA virome throughout the rearing period, as reported by Shah et al. [20], highlights that viral detection at a single time point provides only a snapshot of a continuously evolving community. Age-related changes in the intestinal environment, microbial composition, and immune maturation influence the abundance and diversity of enteric viruses. Consequently, it is important to interpret metagenomic findings with caution and in the context of flock history, management, and concurrent infections.

Next-generation sequencing (NGS) has proven to be a powerful tool for the unbiased detection and characterization of known and novel viruses that cannot be cultured in vitro or propagated in embryonated eggs [13,30,31]. Nevertheless, the mere detection of viral genomes does not imply causation. Several factors can modulate whether an infection remains subclinical or results in overt disease, including the infectious dose, timing of infection, maternal immunity, genetic background of the flock, and immune competence—particularly in relation to immunosuppressive infections such as infectious bursal disease virus (IBDV), chicken anemia virus (CAV), Marek’s disease virus (MDV), or Reticuloendotheliosis virus (REV) [9]. On one broiler farm, Mycoplasma gallisepticum and Mycoplasma synoviae were detected, in addition to infectious coryza (Coryza contagiosa) and Ornithobacterium rhinotracheale (ORT) being present in the flock. The pathogens were identified multiple times using PCR. In a pullet flock, Infectious Bronchitis Virus strain QX was detected by means of RT-PCR. In addition to enteropathogenic viruses and coccidiosis, which were detected in some flocks, mycoplasmas and Avibacterium paragallinarum were the main contributors to poor growth and insufficient uniformity on Farm 1. Whether ORT, which is considered an opportunistic pathogen in broilers, also contributed to the decreased performance on Farm 1 cannot be determined in retrospect. As reported by the attending veterinarian (I.M.), the detection of IBV QX on pullet farm 3 (submission B) likely played only a minor role, as enteritis was far more significant and no IB-typical lesions were found. Of the farms examined, we were able to histologically confirm damage to the immune system. However, an etiological cause could not be determined, as the aforementioned viral pathogens were not included in the testing panel. Nevertheless, it can be assumed that IBDV in particular was circulating on these farms and contributed to the worsening of RSS symptoms. Environmental and management factors such as temperature fluctuations, stocking density, feed composition, water and air quality, or coccidial burden can further exacerbate clinical outcomes [2].

Stressors such as abrupt light changes, prolonged feed deprivation, and rough handling during inspection may also compromise intestinal integrity and immunity, increasing susceptibility to enteric pathogens [9].

Genetic predisposition has been recognized as another determinant influencing susceptibility to MAS and related syndromes. The results of experimental studies have demonstrated differences in disease susceptibility between broiler lines, with White Plymouth Rock line S showing higher sensitivity than Cornish line R [32]. Similarly, Leghorn chickens and turkeys appear less susceptible than fast-growing broilers [33]. These differences may be linked to variations in intestinal morphology, immune cell populations, and cytokine expression profiles during early life [9]. The age of highest susceptibility, typically within the first two weeks of life, coincides with the rapid differentiation of enterocytes in broilers, suggesting that disturbances during this critical phase may predispose birds to malabsorption and enteric disease [9,34]. Histopathological findings of villous atrophy, crypt apoptosis, and heterophil infiltration reported in susceptible lines support the hypothesis that early immune and epithelial responses are crucial determinants of disease outcome [32].

Although astroviruses and Sicinivirus were among the most frequently detected viruses, their role in disease pathogenesis remains to be elucidated. Astroviruses, for instance, are environmentally resilient and resistant to many disinfectants, complicating eradication and increasing the risk of persistence within poultry houses [24]. Therefore, effective biosecurity, longer downtime between production cycles, and thorough cleaning and disinfection are essential preventive measures to reduce infection pressure. With many limitations for such field studies, such as flock and sample sizes, good, well-designed tests and analysis are necessary, and the authors’ future studies should aim to clarify the interaction between different viral agents, host factors, and environmental stressors in the development of enteric disorders. Experimental infection models involving field-relevant broiler lines and co-infections may provide a more comprehensive understanding of the multifactorial mechanisms underlying these complex syndromes.

## 5. Conclusions

The findings of this study demonstrate that enteric viral infections are widespread among poultry flocks in southern Germany and frequently occur as mixed infections involving chicken astrovirus, avian reovirus, and fowl adenovirus. The consistent detection of CAstV and ARV across all investigated flocks, combined with the presence of additional pathogens and concurrent enteric lesions, underscores the multifactorial nature of enteric disease complexes in commercial poultry production. These findings reinforce that no single viral agent can be regarded as the sole causative factor of the observed clinical conditions, but rather that synergistic interactions between viruses, bacteria, parasites, and environmental influences shape disease outcomes.

It was particularly evident on Farm 1 that, in addition to enteropathogenic viruses (CAstV, REO, FAdV-1, and Sicinivirus), coccidiosis also caused intestinal lesions in the broilers. Furthermore, MG, MS, Coryza, and ORT were detected, which additionally impaired their general health status. The multitude of pathogens explains the poor performance and high mortality; implementing a flock health improvement plan is complicated by the fact that the chicks are initially housed in the rearing barn and, from the third to fourth week, in a nearby fattening barn. As a result, two age groups are continuously present on the farm, associated with inadequate biosecurity.

Metagenomic sequencing provided deeper insights into the intestinal virome and enabled the first complete genome assembly of avian Sicinivirus in Germany. Its close phylogenetic relationship to a previously characterized Dutch strain highlights the potential for regional and transboundary spread of emerging enteric viruses within Europe. The identification of nearly identical CAstV and Sicinivirus strains further indicates that these viruses may circulate widely and persistently in poultry populations.

Overall, this work emphasizes the need for enhanced routine surveillance, improved biosecurity strategies, and integrative diagnostic approaches combining classical virology and PCR with next-generation sequencing. Such measures are essential to enhance our understanding of the epidemiology of enteric viruses, mitigate their impact on flock performance, and ultimately support sustainable poultry production.

## Figures and Tables

**Table 1:** In this table, data from seven farms are summarized, categorized by production type (Broilers, Pullets, or Broiler Parents) and management style (Organic vs. Conventional). Data points include bird age (days), population size, and observed clinical symptoms, including gastrointestinal and respiratory distress.

Farm	Type	Husbandry System	Age/Days	Flock Number	Clinical Signs
1	Broilers	Organic	37	15,000	growth problem, severe uniformity, about 25% of the herd are included, bloody diarrhea, and respiratory symptoms
2	Broilers	Organic	14	5000	growth problem, severe uniformity
3	Pullets	Organic	25	20,000	bloody diarrhea, mortalities
4	Broilers	Organic	24	5000	growth problem, severe uniformity, bloody diarrhea
5	Pullets	Conventional	11	6000	mortalities, omphalitis
6	Pullets	Conventional	30	20,000	diarrhea, mortalities
7	Broilers breeder	Conventional	90	2000	growth problem, severe uniformity, bloody diarrhea, mortalities

**Table 2:** Bacteriological Findings: Numbers indicate positive bacteriological results for the specific pathogen (e.g., of 5 dissected birds, 2 *Cl. Perfringens* cultures were positive). The symbols in parentheses represent the semiquantitative growth intensity: 1–4 colonies (+) low, 4–5 colonies (++) moderate, and more than 50 colonies (+++) high.

Farm	Birds Dissected	Culture Method	Organ	*E. coli*	*Cl. perfringens*	*G. anatis*	*E.* *Gallinarum*	*E.* *faecalis*	*E.* *fergusonii*	*E.* *durans*	*S.* *chromogenes*	Neg	Unsp
1	5	aerobic	Liver	1x (++)						1x (++)	1x (++)		
		aerobic	Small Intestine										2x
		anaerobic	Liver		2x (−)								
		anaerobic	Small Intestine		3x (−)								
2	7	aerobic	Liver										3x
3	12	aerobic	Liver	3x (++–+++)		1x (+)							1x
		aerobic	Small Intestine									2x	2x
		anaerobic	Liver		1x (+)							2x	
		anaerobic	Small Intestine									2x	2x
4	4	aerobic	Yolk Sack	1x (++)			1x (++ –+++)	1x (+++)					
		aerobic	Small Intestine										2x
		anaerobic	Liver		1x (++)								
		anaerobic	Small Intestine		1x (+)							1x	
5	4	aerobic	Liver			1x (+)							1x
		aerobic	Spleen			1x (+)							1x
		aerobic	Small Intestine										2x
		anaerobic	Liver									2x	
		anaerobic	Spleen									2x	
		anaerobic	Small Intestine									2x	
6	5	aerobic	Liver	1x (+)		2x (+)			1x (++)				
		aerobic	Small Intestine										2x
		anaerobic	Liver									2x	
		anaerobic	Small Intestine									2x	

**Table 3:** In this table, the detection of coccidia in necropsied birds is summarized. The results demonstrate the intensity of coccidial lesions in the small intestine and ceca. Lesion severity was assessed based on gross pathology and histopathology and classified as mild, moderate, or severe.

Farm	Birds Dissected	Organ	Coccidia
1	5	Ceca	Moderate
		Small Intestine	Mild
3	12	Ceca	Mild to moderate
		Small Intestine	Mild
5	4	Intestine	Negative
6	5	Intestine	Moderate
Farm	Birds dissected	Organ	Coccidia
1	5	Caeca	moderate
		Small Intestine	mild
3	12	Caeca	mild to moderate
		Small Intestine	mild
5	4	Intestine	negative
6	5	Intestine	moderate

**Table 4:** Summary of viral screening results for CAstV, ARV, and FAdV-1 A–E on seven poultry farms. Viral prevalence was assessed across broilers, pullets, and broiler parents. Numerical values represent Ct scores for CAstV and AVR, where “neg” indicates no detection. FAdV-A-E status is reported as positive (pos) or negative (neg).

Farm	Type	CAstV	ARV	FAdV-1A-E
1	Broilers	20.7	29.1	pos
2	Broilers	30.1	34.1	neg
3	Pullets	35.3	33.4	neg
4	Broilers	27.9	29	pos
5	Pullets	32	24.6	neg
6	Pullets	36.4	neg	pos
7	Broilers breeder	31.2	29.2	pos

## Data Availability

All data generated or analyzed during this study are included in this article. The relevant accession numbers for sequence data that have been deposited in GenBank are PX395415–PX395426 for CAstV and PX068467 for avian Sicinivirus.

## Abbreviations

The following abbreviations are used in this manuscript:

ANV	Avian Nephritis Virus
ARV	Avian Reovirus
FAdV-1	Fowl Adenovirus-1
IB	Infectious Bronchitis
MAS	Malabsorption Syndrome
MDV	Marek’s Disease Virus
NGS	Next-Generation Sequencing
ORT	Ornithobacter rhinotracheale
REV	Reticuloendothelosis Virus
RSS	Runting–Stunting Syndrome
RV	Rotavirus
